# Bayesian non-parametrics and the probabilistic approach to modelling

**DOI:** 10.1098/rsta.2011.0553

**Published:** 2013-02-13

**Authors:** Zoubin Ghahramani

**Affiliations:** Department of Engineering, University of Cambridge, Cambridge CB2 1PZ, UK

**Keywords:** probabilistic modelling, Bayesian statistics, non-parametrics, machine learning

## Abstract

Modelling is fundamental to many fields of science and engineering. A model can be thought of as a representation of possible data one could predict from a system. The probabilistic approach to modelling uses probability theory to express all aspects of uncertainty in the model. The probabilistic approach is synonymous with Bayesian modelling, which simply uses the rules of probability theory in order to make predictions, compare alternative models, and learn model parameters and structure from data. This simple and elegant framework is most powerful when coupled with flexible probabilistic models. Flexibility is achieved through the use of Bayesian non-parametrics. This article provides an overview of probabilistic modelling and an accessible survey of some of the main tools in Bayesian non-parametrics. The survey covers the use of Bayesian non-parametrics for modelling unknown functions, density estimation, clustering, time-series modelling, and representing sparsity, hierarchies, and covariance structure. More specifically, it gives brief non-technical overviews of Gaussian processes, Dirichlet processes, infinite hidden Markov models, Indian buffet processes, Kingman’s coalescent, Dirichlet diffusion trees and Wishart processes.

## 1. Introduction

Modelling is central to the sciences. Models allow one to make predictions, to understand phenomena, and to quantify, compare and falsify hypotheses.

Modelling is also at the core of intelligence. Both artificial and biological systems that exhibit intelligence must be able to make predictions, anticipate outcomes of their actions and update their ability to make predictions in light of new data. It is hard to imagine how a system could do this without building models of the environment that the system interacts with. It is thus not surprising that many theories in cognitive science are based around the idea of building internal models [1,2,3].

A model is simply a compact representation of possible data one could observe. As such, it may be more interpretable than the observed data itself, providing a useful representation of data. A model must be able to make forecasts of possible future data; otherwise, it seems impossible to falsify a model in light of new data. I will use the term *forecast* in a very general way to refer to the process of making any claims about unobserved data on the basis of observed data; I will also use *predict* interchangeably with forecast. For all non-trivial phenomena, forecasts have to include some representation of the forecasting uncertainty. Deterministic forecasts (e.g. tomorrow’s high temperature *will be* 17^°^C) are too brittle and therefore easy to falsify.

Ideally, a model should also be adaptive. By *adaptive*, I mean that the forecasts of the model should change, depending on the data observed so far. Such adaptation, or learning, should hopefully have the effect of making the model’s forecasts be better aligned with actual data. For example, if the forecasts are in the form of probability distributions, adaptation should have the effect that the forecast probability assigned to what actually happens should increase after the model has seen more data, although this cannot be generally guaranteed.

It is clear that all forecasts need to represent uncertainty. Observable data are generally corrupted by noise in the measurement process, and this noise needs to be incorporated in the forecast uncertainty. But uncertainty lurks at many other levels in any model. The amount of noise in the measurement process may itself be unknown. The model may have a number of parameters that are unknown. The structure of the model itself may be uncertain, or there may be multiple plausible competing models for the data. The forecasting system should ideally produce forecasts that incorporate all reasonable sources of uncertainty. As we will see, the Bayesian framework provides a natural and coherent approach for representing and manipulating all forms of uncertainty in modelling.

## 2. The Bayesian framework

The fundamental idea in Bayesian modelling is to use the mathematics of probability theory to represent and manipulate all forms of uncertainty in the model. This is a surprisingly simple yet powerful idea.

The good news is that there are only two rules of probability theory one needs to remember: the sum rule and the product rule.^1^ Consider a pair of random variables *x* and *y* taking on values in some spaces 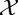 and 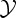, respectively. The sum rule states that if I know the joint probability of two random variables, *x* and *y*, I can obtain the marginal probability of *x* by summing over all possible values of *y*,

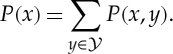

If *y* is continuous, we simply replace the sum with an integral. If I have a model for the joint probability distribution of the high temperature in London and Cambridge, I can obtain the marginal distribution for the high temperature in Cambridge summing out London’s temperature.

The product rule states that the joint probability of *x* and *y* can be decomposed into the product of the marginal probability of *x* and the conditional probability of *y* given *x* (or the other way around),

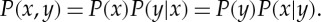

Combining the sum and product rule and rearranging a bit, we obtain Bayes rule as a corollary,

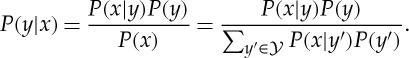



Let us now use these equations in a canonical modelling context. We will use 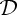 to represent the observable data. Often we talk about the data being a *set* consisting of a number of ‘data points’ or measurements, 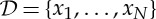, but this is in no way a necessary assumption. For example, the data could be a single image, an observed graph or an ordered *sequence* of measurements rather than a set. Our model will be indexed by *m* and we may want to consider multiple alternative models, e.g. *m*, *m*′, etc. Each model usually has a number of free parameters, which we will denote with *θ*, which can be a vector if needed.

First we need to make sure that the model *m* is well defined, in the sense that it can predict or forecast data. As previously discussed, we use probability theory to represent the model forecasts. For any given setting of the model parameters, the model must be able to produce a forecast of the form

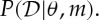

This probability of the data as a function of the parameters is the *likelihood* of the parameters. With the likelihood, for any given setting of the parameters, we are in the position of making forecasts. However, the model *m* is not fully defined until we specify a ‘range’ for the parameter values, *θ*. A linear regression model that states that the slope can take values between −1 and +1 is a very different model from one that specifies that the slope can take values between −100 and +100. In fact, to fully specify a model, we need to do a bit more than specify the range of parameters, we need to define a distribution over this range. Only then will our model *m* be able to make forecasts. We see this by writing the forecast probability of the model using the sum and product rules,
2.1
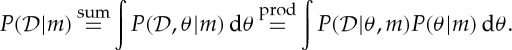

The expression 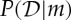 is variously called the *marginal likelihood*, *model evidence* or *integrated likelihood*. The *prior* over the parameters, *P*(*θ*|*m*), plays the role of specifying the range as described above (e.g. it could be uniform on [−1,+1]) in the form of a distribution over the allowable values of the parameters. Without a prior, our model is not well defined: we cannot generate or forecast data until we know how to choose values for *θ*.^2^ Once the prior and likelihood are defined, and only then, is the model *m* fully specified in the sense that it can *generate possible datasets*.

People often object to Bayesian methods on the basis that it forces one to define priors on the parameters. This, in my opinion, is completely misguided. All models make assumptions; without assumptions, it is impossible to make any forecasts or predictions from observed data. The first stage of the Bayesian modelling framework is to explicitly state all assumptions using the language of probability theory. Specifying both the prior and likelihood is a necessary requirement so that the model is well defined. In fact, the distinction between prior and likelihood is arbitrary^3^ ; both are essential parts of the *model*.

People also object to the use of priors on the parameters on the grounds that they do not think of the parameters as being ‘random’ variables. For example, if one is estimating the mass of a planet from astronomical data, the mass of the planet is not ‘random’ in the colloquial sense of the word.^4^ This is a misunderstanding of the semantics of probabilities in Bayesian modelling. Probabilities are used to represent our *uncertainty* about unknown quantities. They are just as good at modelling uncertainty for repeatable experiments such as the outcome of a roll of a die, as they are for modelling uncertainty in the mass of a planet. Incidentally, both forms of uncertainty are fundamentally subjective; one’s uncertainty about a roll of a die depends on one’s knowledge of the exact initial conditions of the roll, in just the same way as the uncertainty about the mass of the planet depends on knowledge of the observational data on the planet’s orbit.

Finally, people trained in the sciences are uncomfortable with the very notion of subjectivity in data analysis and modelling. This again is deeply misguided: all models involve assumptions, and all conclusions drawn from data analysis are conditional on assumptions. The probabilistic framework *forces* the scientist to be completely transparent about the assumptions, by expressing all assumptions as distributions on unknown quantities. These assumptions can then be easily contested, and the same data can be reanalysed under different modelling assumptions (and priors). The fact that conclusions could change depending on the assumptions is essential to good scientific practice. Fortunately, given enough data, the effect of the prior is generally overcome by the likelihood, and posterior conclusions will converge [4,5,6]. This is directly analogous to the progress of science, where of many possible hypotheses, only the ones consistent with the data will survive. Bayesian modelling is *subjective* but not *arbitrary*: given a full specification of the model, and the data, there is only one way to reason about any quantity of interest.

Modelling thus becomes a very simple procedure:

— write down your assumptions (possible models, parameters, noise processes, etc.), representing all forms of uncertainty using the language of probability theory and
— given the data, use probability theory to make inferences about any unknown quantities in your model, or to make predictions from the model.

This process lends itself very naturally to a sequential processing of data. Your posterior after observing some data 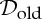, 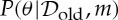 is your prior before observing new data, 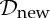,

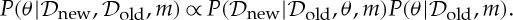



The sum and product rule also tell us how to make predictions from a model. Consider predicting some unknown quantity *x* (e.g. the next data point) given observed data 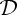 and model *m*,
2.2
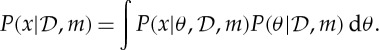

This is a very intuitively satisfying expression which tells us that our predictions or forecasts are a weighted average of the forecasts from different parameter values, weighted by the posterior probability of each parameter value given the data observed so far. For parametric models, this simplifies because *given* the parameters forecasts are independent of the observed data: 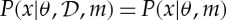. We will revisit this point as we discuss parametric versus non-parametric models in §3. If we are considering a number of models *m*, *m*′, etc., then by the sum and product rules, our forecasts are an average over models weighted by their posteriors.

The probabilistic modelling framework also provides intuitive answers to problems in model comparison. Assuming a set of competing probabilistic models 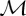, given some observed data, we can evaluate the posterior probability of a particular model *m*,

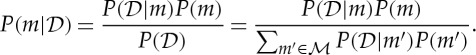

Note the prominent role played by the marginal likelihood, 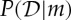.

**Figure 1.**
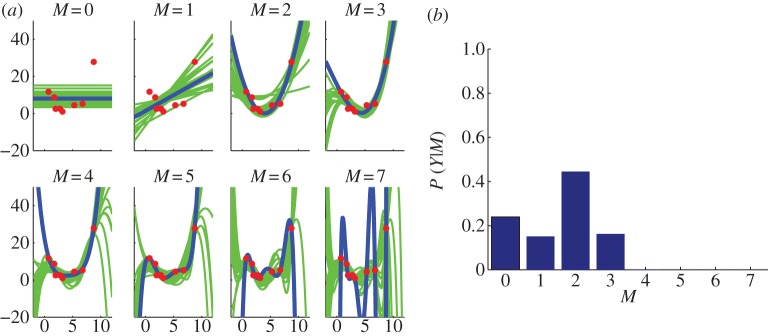
Marginal likelihoods, Occam’s razor and overfitting: consider modelling a function *y*=*f*(*x*)+*ϵ* describing the relationship between some input variable *x*, and some output or response variable *y*. (*a*) The red dots in the plots on the left-hand side are a dataset of eight (*x*,*y*) pairs of points. There are many possible *f* that could model this given data. Let us consider polynomials of different order, ranging from constant (*M*=0), linear (*M*=1), quadratic (*M*=2), etc., to seventh order (*M*=7). The blue curves depict maximum-likelihood polynomials fit to the data under Gaussian noise assumptions (i.e. least-squares fits). Clearly, the *M*=7 polynomial can fit the data perfectly, but it seems to be overfitting wildly, predicting that the function will shoot off up or down between neighbouring observed data points. By contrast, the constant polynomial may be underfitting, in the sense that it might not pick up some of the structure in the data. The green curves indicate 20 random samples from the Bayesian posterior of polynomials of different order given this data. A Gaussian prior was used for the coefficients, and an inverse gamma prior on the noise variance (these conjugate choices mean that the posterior can be analytically integrated). The samples show that there is considerable posterior uncertainty given the data, and also that the maximum-likelihood estimate can be very different from the typical sample from the posterior. (*b*) The normalized model evidence or marginal likelihood for this model is plotted as a function of the model order, *P*(*Y* |*M*), where the dataset *Y* are the eight observed output *y* values. Note that given the data, model orders ranging from *M*=0 to *M*=3 have considerably higher marginal likelihood than other model orders, which seems plausible given the data. Higher-order models, *M*>3, have relatively much smaller marginal likelihood, which is not visible on this scale. The decrease in marginal likelihood as a function of model order is a reflection of the automatic Occam razor that results from Bayesian marginalization.

**Figure 2.**
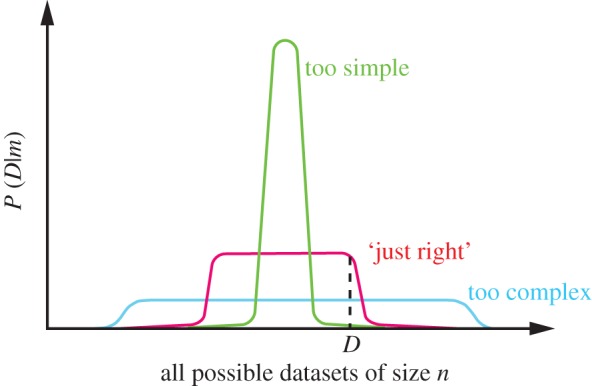
An illustration of Occam’s razor. Consider all possible datasets of some fixed size *n*. Competing probabilistic models correspond to alternative distributions over the datasets. Here, we have illustrated three possible models that spread their probability mass in different ways over these possible datasets. A *complex* model (shown in blue) spreads its mass over many more possible datasets, whereas a *simple* model (shown in green) concentrates its mass on a smaller fraction of possible data. Because probabilities have to sum to one, the complex model spreads its mass at the cost of not being able to model simple datasets as well as a simple model—this normalization is what results in an automatic Occam razor. Given any particular dataset, here indicated by the dotted line, we can use the marginal likelihood to reject both overly simple models, and overly complex models. This figure is inspired by a figure from MacKay [10], and an actual realization of this figure on a toy classification problem is discussed in Murray & Ghahramani [11].

Importantly, this marginal likelihood captures a preference for simpler models known as Bayesian Occam’s razor [7,8,9]. Consider a set of nested models, for example, different order polynomials (e.g. constant, linear, quadratic, cubic, etc.) used to fit some regression relationship (figure 1). Clearly, a higher-order model such as the cubic polynomial is strictly more complex than a lower-order model such as the linear polynomial. Model fitting procedures based on optimization (such as maximum-likelihood methods or penalized-likelihood methods) need to take great care not to *overfit* the data by fitting the parameters of an overly complex model to a relatively small dataset. Overfitting is not a problem for fully Bayesian methods, as there is no ‘fitting’ of the model to the data. We have only the sum rule and the product rule to work with, there is no ‘optimize’ rule in probability theory. A more complex model, say one that has more parameters, simply spreads its predictive probability mass over more possible datasets than a simpler model. If all models are specified as probability distributions over datasets, because probability distributions have to sum to one, all models have the same amount of probability mass to spread over possible data (figure 2). Given a particular dataset, it therefore becomes possible to reject both models that are too simple or too complex simply by using the rules of probability theory.

This approach to Bayesian model comparison can be used to solve a vast range of problems in learning the structure of complex models. For example, it has been used to learn the number of clusters in a mixture model [12,13], finding relevant variables or features in a prediction problem [14,15], discovering the number of states in a hidden Markov model (HMM) [16] and learning the dependency structure between variables in a probabilistic graphical model [17].

The above approach to model comparison relies on the ability to enumerate a set of models 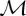 to be compared. This is often reasonable in scientific settings where there are a number of competing hypotheses to explain some phenomenon. Bayesian Occam’s razor ensures that overly complex models are adequately penalized when doing model comparison. However, the complexity of real-world phenomena often requires us to consider complex models that are flexible enough to capture the structure in real data. Flexible models are not only more realistic, but will also generally result in more reasonable forecasts than simpler models. Bayesian non-parametrics provides a natural framework for defining flexible models.

## 3. Non-parametric models

The best way to understand non-parametric models is by first reminding ourselves of what parametric models are. A parametric model has some finite set of parameters *θ*. Given these parameters, future predictions, *x*, are independent of the observed data,

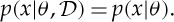

The parameters therefore capture everything there is to know about the data that is relevant for predicting future data.

We can think of all models as *information channels* from past data 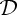 to future predictions *x*. The parameters in a parametric model constitute a bottleneck in this information channel. The complexity of the model, and the capacity of the channel, is bounded, even if the amount of observed data becomes unbounded. Parametric models are therefore not generally very flexible.

By contrast, a *non-parametric* model assumes that the data distribution cannot be defined in terms of such a finite set of parameters. However, often we can think of non-parametric models as being defined in terms of an infinite-dimensional *θ*. More formally, the infinite-dimensional *θ* is often represented as a function. The term *non-parametric* is therefore a bit of a misnomer; it is not that non-parametric models do not have parameters; in fact, they have infinitely many parameters. Because of this, non-parametric models cannot be explicitly represented in terms of their parameters.

From the information channel viewpoint, we have removed the bottleneck. The amount of information that *θ* can capture about the data 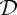 grows as the amount of data grows. This makes non-parametric models more flexible than parametric models.^5^

There is another way to view the difference between parametric and non-parametric models. Predictions from a parametric model are explicitly and compactly summarized through the parameters *θ*, *P*(*x*|*θ*). Non-parametric models, by contrast, cannot be summarized in this way. Because of this, predictions from a non-parametric are necessarily *memory-based*, 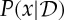; to make predictions, we need to store or remember a growing amount of information about the training data, 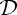.

Non-parametric models are inextricably tied to the notion of *exchangeability*. A sequence is exchangeable if its joint distribution is invariant under arbitrary permutation of the indices. Consider modelling a dataset {*x*_1_,…,*x*_*N*_} under the assumption that the ordering of the elements is uninformative. This dataset may be a collection of documents, images or any other sort of object collected in a manner such that either the ordering is irrelevant or completely unknown.^6^ De Finetti’s theorem states that a sequence is exchangeable if and only if there exists some *θ* such that the elements *x*_*n*_ are independently and identically distributed (iid) from some unknown distribution indexed by *θ* [19]. Importantly, *θ* may need to be infinite dimensional as it needs to index the space of probability measures (non-negative functions that normalize to one).

The consequence of de Finetti’s theorem is that if we want to model exchangeable data in full generality, we need to consider putting distributions on unknown probability measures.

Distributions on measures, functions and other infinite-dimensional objects are thus central to Bayesian non-parametric modelling. Many of these distributions are infinite-dimensional versions of their finite-dimensional counterparts, and in fact a good strategy for deriving Bayesian non-parametric models is to start from a parametric model and ‘take the infinite limit’ [20]. Distributions on infinite-dimensional objects are the main subject of study in stochastic process theory, and therefore much of the terminology used in Bayesian non-parametrics is borrowed from this field.^7^

Two of the classical building blocks for Bayesian non-parametric models are the Gaussian process (GP) and the Dirichlet process (DP). I will give an overview of these models in §§4 and 5, with an emphasis on their applications to general problems in machine learning and statistics, including regression, classification, clustering and density estimation (these problems will also be described in those sections). I will also cover newer building blocks that can represent non-parametric distributions over time series, sparse matrices, hierarchies, and covariances (§§6–9). In order to give a broad overview, I will necessarily have to avoid going in much depth into any of the specific topics, providing instead pointers to the relevant literature. Admittedly, my overview is based on my personal view of the field, and is therefore biased towards areas of Bayesian non-parametrics to which my colleagues and I have contributed, and misses other important areas.

## 4. Modelling functions, classification and regression: Gaussian processes

GPs are a distribution over functions that can be used in numerous contexts where one’s model requires one to represent an unknown function [21]. One-dimensional GPs indexed by time are familiar to many fields: Brownian motion, Wiener processes, Ornstein–Uhlenbeck processes, linear Gaussian state-space models, and many random walks are all examples of GPs. For historical reasons, GPs are also sometimes associated with spatial statistics, for example, modelling temperature as a function of spatial location. Within machine learning, GPs are often used for nonlinear regression and classification.

Consider the following simple model for nonlinear regression:

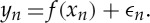

Here, we wish to model the relationship between an input (or covariate) *x* and an output (or response variable) *y*. The subscript *n* indexes data points in our dataset 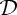, *ϵ*_*n*_ is some additive noise and crucially, *f* is the unknown regression function we wish to learn about from data.

Assume we have a distribution over functions *f* and we evaluate the marginal distribution it assigns to the vector ***f***=(*f*(*x*_1_),…*f*(*x*_*N*_)). If, for any choice of input points, (*x*_1_,…,*x*_*N*_), the marginal distribution over ***f*** is multi-variate Gaussian, then the distribution over the function *f* is said to be a GP. In other words, a GP is an infinite-dimensional generalization of the multi-variate Gaussian. Analogous to the Gaussian, a GP is parametrized by a mean function, and a covariance function.

The application of GPs to regression is straightforward. Starting with a prior on functions *p*(*f*), we condition on the data to obtain a posterior 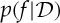. When the noise *ϵ* is assumed to be Gaussian, all computations reduce to operations on *N*-dimensional Gaussians. The infinitely many other dimensions of *f* can be marginalized out analytically.

Classification problems correspond to predicting categorical response or output variables, e.g. *y*∈{cat,dog}. GP regression can be modified to do classification simply by introducing a link function that maps the real values *f*(*x*) into probabilities over the classes. Computing 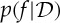 exactly becomes intractable, but many good methods exist for approximating the required integrals (§10).

## 5. Density estimation and clustering: Dirichlet processes and Chinese restaurant processes

We now consider two distinct problems—density estimation and clustering—and describe a close link between the two when approached from a Bayesian non-parametric modelling approach.

 *Density estimation* refers to the problem of inferring an unknown density *p* from data 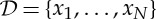. Let us first consider a very simple situation where the data points belong to a discrete and finite space with *K* possible values, 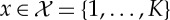. Any distribution on 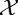 can be represented by a *K*-dimensional non-negative vector ***p*** that sums to one. To infer ***p*** from 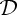, we need a reasonable prior on finite distributions. The Dirichlet distribution is a natural choice that takes the form

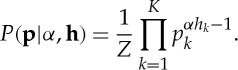

Here, *Z* is a normalizing constant, ***h*** is the mean of ***p*** and *α*>0 controls the dispersion around the mean. A very attractive property of the Dirichlet distribution is that it is *conjugate* in the sense that the posterior 
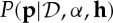
 is also Dirichlet. Another nice property is that for all but pathological choices of the parameters, it has *good coverage* in the sense that it puts some non-zero probability mass near all possible values of ***p***.

The DP extends the Dirichlet distribution to countable or uncountably infinite spaces 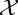. It has the property that for any *finite* partition of 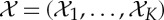, it marginalizes to a *K*-dimensional Dirichlet distribution on the measure (i.e. mass) assigned to each element of the partition [22]. We write that the probability measure *G* is drawn from a DP prior as *G*∼DP(*α*,*H*) analogous to our notation for the Dirichlet distribution. An excellent introduction to the DP is provided by Teh [23].

Conjugacy and good coverage suggest that the DP could be a very good general purpose non-parametric density estimator. Unfortunately, the distributions drawn from a DP prior are, with probability one, discrete so they do not have a density. In fact, a draw from a DP prior can be represented in the following way:
5.1
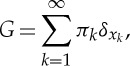

where the sum is an infinite sum, the *π*_*k*_ are masses that sum to one, *δ* is the Dirac-delta function and the *x*_*k*_ are locations for those probability masses drawn iid from the base measure *H*, which controls the mean of *G*. To alleviate the problem of discreteness, the DP is often used as a prior on the parameters of a mixture model, with the whole model now called a Dirichlet process mixture (DPM) [24,25,26,20].

The particular example of a DPM of Gaussians, also known as an infinite Gaussian mixture model [27], is widely used for both density estimation and clustering. *Clustering* refers to the problem of finding groupings of objects or data points such that similar objects belong to the same group and dissimilar objects belong to different groups. An example application of clustering is finding groups of similar celestial objects in a large astronomical survey. Posed abstractly in this way, clustering is not a well-defined problem (how many groups should we find? What does ‘similar’ mean?). However, if we restrict ourselves to assuming that each cluster can be captured by some parametrized probability distribution over data points, such as a Gaussian, then clustering becomes a well-defined probabilistic inference problem. Bayesian non-parametric clustering using DPMs is a natural extension of classical clustering algorithms such as the Expectation Maximization algorithm for finite mixture models or k-means [28]. In a finite mixture model, the data are assumed to come from a distribution composed of *K* components,
5.2
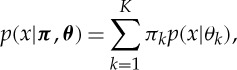

with mixing weights ***π*** that sum to one, and parameters *θ*_*k*_ for each component. An infinite mixture model considers the limit of 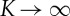, and has the property that it allows the number of observed clusters to grow with the number of observed data points. A DPM can be obtained as an infinite limit of a finite mixture model in many ways, but let us consider the following construction:

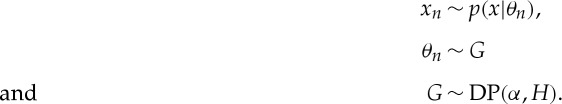

As *G* is discrete, the values of *θ*_*n*_ will repeat, which results in a clustering of the data points. By equation (5.1), the *π*_*k*_ correspond to the mixing weights of the infinitely many clusters, which can be compared with the finite counterpart (equation (5.2)). The distribution over partitions of the data points induced by the DPM is known as a *Chinese restaurant process* (CRP; Aldous [29]).

The DPM, apart from being mathematically elegant, has some practical advantages over traditional clustering algorithms. There can be computational advantages to running a single instance of a DPM inference algorithm that automatically infers the number of clusters, rather than having to run multiple instances of an algorithm to compare different hypotheses on the number of clusters. Moreover, at test time, the DPM always allows for the possibility that a new test point (e.g. a new astronomical object) belongs to a cluster that was not observed in the training data. This makes DPM predictions somewhat more robust to outliers.

## 6. Discrete state time series: infinite hidden Markov models

Many processes of interest produce sequential data for which models that assume exchangeability are inadequate. For example, natural language text, protein amino acid sequences, financial time series and natural sounds all have sequential structure that is important to model.

One of the most widely used models for times series is the HMM. An HMM assumes that a series of observations (*x*_1_,…,*x*_*T*_) was generated by a process that can be in one of *K* different discrete states at each point in time, *s*_*t*_∈{1,…,*K*}. Moreover, in an HMM, *s*_*t*_ fully captures the state of the system at time *t* in the sense that given *s*_*t*_ the future evolution of the system does not depend on the state at times previous to *t*; this is the *Markov property*: *P*(*s*_*t*+1_|*s*_1_,…,*s*_*t*_)=*P*(*s*_*t*+1_|*s*_*t*_). Finally, the observations are assumed to be drawn independently given the hidden states, through an emission process *P*(*x*_*t*_|*s*_*t*_).

An example of an interesting application of HMMs in the physical and biological sciences is the modelling of single ion channel kinetics [30]. Here, the measured time series of currents from an ion channel are modelled by assuming that at each point in time the channel macromolecule can be in one of many different conformational states, and that there is a transition matrix defining the probability of transitioning between each of these states.

Learning an HMM involves inferring both parameters of the transition process *P*(*s*_*t*+1_|*s*_*t*_), which is in general a *K*×*K* transition matrix, and parameters of the emission process, *P*(*x*_*t*_|*s*_*t*_) [31]. The problem of learning the structure of an HMM corresponds to inferring the number of hidden states, *K*, from data. Rather than doing model selection over varying *K*, we would like to develop a non-parametric approach to HMMs where the model has countably infinitely many hidden states at its disposal. This can be useful both when we do not believe that any finite HMM can capture the data generating process well, and in situations where we believe that the data was actually generated from a finite-state process, but we simply do not know how many states this process should have.^8^

The key insight that allows one to develop a non-parametric HMM is that the finite HMM is a time-series generalization of finite mixture models. At each time step, the HMM assumes that the observation *x*_*t*_ was generated by one of *K* mixture components, where *s*_*t*_ indicated the component (or cluster) used. The only difference between HMMs and mixture models is that the mixture indicators in an HMM depend on the ones at the previous time step.

Using this insight, Beal *et al.* [32] developed the infinite HMM (iHMM). The basic idea was to consider a Bayesian HMM with countably infinitely many states. The main difficulty was to define a sensible prior on the parameters of the 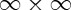 transition matrix. In the finite *K*-dimensional case, one would typically use independent symmetric Dirichlet prior distributions for each row of the transition matrix (where a *k*th row corresponds to the vector of all outgoing transition probabilities from state *k*). In the infinite limit, the independent Dirichlet prior does not result in a sensible model, as under this prior, with probability one, the HMM will keep transitioning to new states rather than revisiting previous states. The solution developed in Beal *et al.* [32] was to couple the rows by using a hierarchical DP, a solution analogous to a reinforced urn process in probability theory [33]. This work was followed up in the elegant paper by Teh *et al.* [34], which further developed the hierarchical DP and proposed an improved Markov chain Monte Carlo (MCMC) sampler for the iHMM.

Since the original paper, there have been a number of conceptual and algorithmic developments of the iHMM. The beam sampler provides an efficient way of sampling the iHMM by using dynamic programming forward–backward style message passing [35].^9^ Parallel and distributed implementations of the iHMM allow larger scale deployments [36]. The block diagonal iHMM is an extension that groups the hidden states into clusters of states, effectively hierarchically partitioning the state space [37]. The iHMM can be extended to have a power-law structure on the hidden states by using the Pitman–Yor process [38,39] and has been successfully applied to diverse problems such as language modelling [39] and speaker diarization [40].

## 7. Sparse matrices and overlapping clusters: Indian buffet processes

One limitation of DPMs, the iHMM and clustering models in general is that each data point is modelled as belonging to one of a set of mutually exclusive clusters. Although the non-parametric variants are flexible, in that they allow countably infinitely many clusters, they do not allow a data point to belong to multiple clusters at the same time—they fundamentally define distributions over *partitions* of the data.

We would like building blocks for our models that can allow overlapping cluster membership. For example, to understand a person’s network of friendships, we really need models that can capture the idea that a person can belong *simultaneously* to many possible social groupings based on workplace, family, housing location, high school, hobbies, etc. This type of hidden structure in data is sometimes called *factorial* structure [41,42,43].

The *Indian buffet process* (IBP; [44,45]) is a probabilistic object that can be used to represent non-parametric factorial structure. There are a number of ways of understanding and deriving the IBP. Let us start with a sparse binary matrix view of IBPs.

Consider an *N*×*K* binary matrix, *Z*, where we can think of the rows of the matrix as corresponding to objects or data points, and the columns as features or clusters. An element of this matrix *z*_*nk*_=1 may denote that object *n* possesses hidden feature *k* (or belongs to cluster *k*), and features (clusters) are not mutually exclusive in that an object can have multiple features. We wish to define a very simple distribution that is exchangeable over the objects (rows). A very simple choice is to assume that the columns are independent. We can therefore model each column through a single unknown parameter, *θ*_*k*_, representing the frequency of feature *k*, *p*(*z*_*nk*_=1|*θ*_*k*_)=*θ*_*k*_. Full specification for this model requires some choice for the prior distribution of *θ*_*k*_. A natural choice is the beta distribution (the special case of a Dirichlet when there are only two outcomes) that happens to be conjugate to the Bernoulli likelihood, allowing *θ*_*k*_ to be integrated out.

Like in the previous cases, we wish to consider models with infinitely many possible features or clusters, so we therefore have to examine the limit 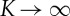. The beta distribution has two parameters, *α*, *β*, and the mean of the beta distribution is *α*/(*α*+*β*). For fixed *α*,*β* and *N* in the limit 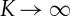, the matrix *Z* will have infinitely many ones in it, which makes it computationally and statistically of limited interest. However, consider scaling the first parameter of the beta distribution and setting the second parameter to one, i.e. using a beta(*α*/*K*,1) prior for each *θ*_*k*_.^10^ In this case, the limit on the distribution of *Z* has a number of nice properties: (i) the number of ones in each row is distributed as Poisson(*α*), (ii) the total expected number of ones is *αN*, (iii) the number of non-zero columns grown as 
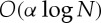
, and (iv) the rows are exchangeable. This distribution is the IBP.

**Figure 3.**
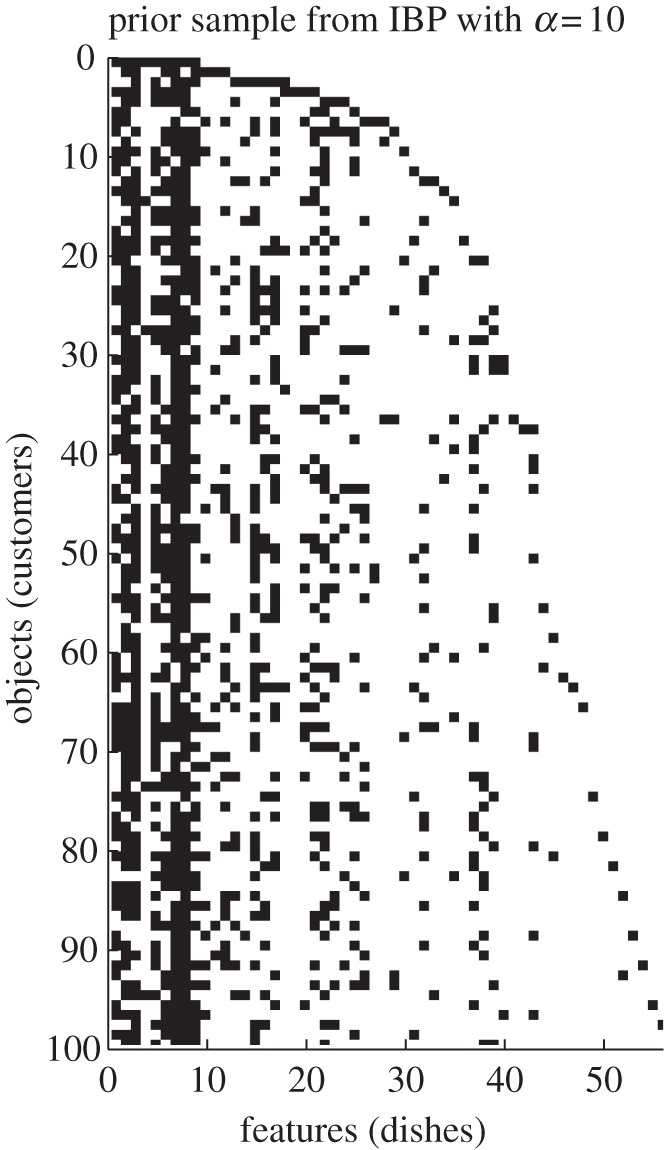
A sample from an IBP matrix, with columns reordered. Each row has, on average, 10 ones. Note the logarithmic growth of non-zero columns with rows. For the ‘restaurant’ analogy where customers enter a buffet with infinitely many dishes, you can refer to the original IBP papers.

Note that the distribution described earlier has infinitely many columns of zeros. Because the columns are all iid, once we sample a matrix, we can reorder its columns into a more usable representation such as the one shown in figure 3. In many applications, we are specifically interested in sparse binary matrices; for example, to represent which factors are non-zero in sparse latent factor models [46], for binary matrix factorization [47] or to represent the adjacency matrix in a graph [48]. However, it is sometimes useful to view the IBP as a more abstract probabilistic object. Whereas the CRP is an infinitely exchangeable distribution over *partitions* of a set of objects, the IBP is an infinitely exchangeable distribution over *subsets* of a set of objects. Each object belongs to Poisson(*α*) subsets (or clusters), and as *N* increases, both the number of subsets grows (logarithmically) and the size of each subset grows (linearly) with *N*.

Since its introduction, many interesting properties and extensions of the IBP have been uncovered. Because the IBP defines an exchangeable distribution, it has a de Finetti mixing distribution; for the IBP, Thibaux & Jordan [49] showed that this is the beta process [50]. An important extension of the IBP is the three-parameter model that exhibits a power-law growth in the number of clusters [51].

Non-parametric models that use the IBP to define sparse latent variables have been applied to a number of different problems, as reviewed in Griffiths & Ghahramani [45]. A very interesting application of the IBP is to the problem of *network modelling:* modelling the connections between objects or entities in social, biological and physical networks [52]. While many models of networks are based on the idea of discovering communities or clusters of the nodes, the IBP allows each node to belong to multiple overlapping communities or clusters, a property that can be exploited to obtain improved predictive performance in tasks such as link prediction [53,54,55].

**Figure 4.**
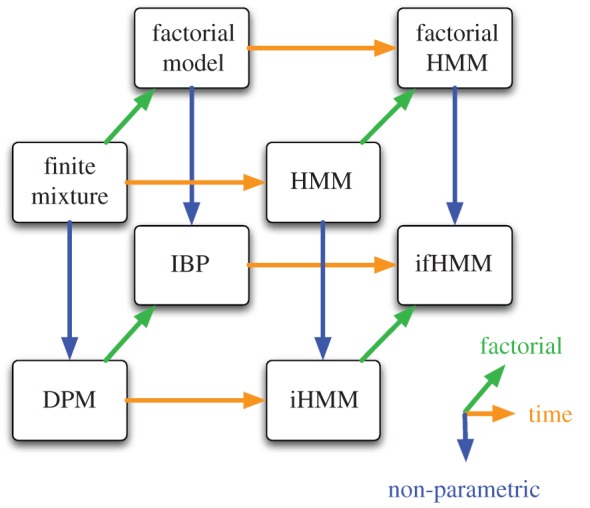
A diagram representing how some models relate to each other. We start from finite mixture models and consider three different ways of extending them. Orange arrows correspond to time-series versions of static (iid) models. Blue arrows correspond to Bayesian non-parametric versions of finite parametric models. Green arrows correspond to factorial (overlapping subset) versions of clustering (non-overlapping) models. ifHMM, infinite factorial hidden Markov model.

Figure 4 shows a useful visualization of the relationship between a number of models. Here, we can see that the DPM (§5), the iHMM (§6) and the IBP (§7) can all be related to each other. Combining the key features of all models results in the infinite factorial HMM, a Bayesian non-parametric time-series model with factorial hidden structure [56].

## 8. Hierarchies: Kingman’s coalescent, and Dirichlet and Pitman–Yor diffusion trees

It is useful to distinguish between *flat* clustering models and *hierarchical* clustering models. In the former, data are partitioned into clusters, while in the latter, these clusters in turn are also partitioned into superclusters in a hierarchical manner. This results in a *tree* (or dendrogram) where at the top level, we have a single root node corresponding to one coarse cluster with all the data points, while at the bottom, we have leaves corresponding to fine clusters with a single data point in each cluster [28].

Such hierarchical clustering models are useful for many reasons. First, many natural phenomena are well modelled through hierarchies. For example, the evolution of biological organisms results in phylogenetic trees that are well modelled by a hierarchy, and real-world objects can often be decomposed into parts, subparts, etc. Second, hierarchies allow one to tie together parameters of complex models so as to improve generalization in learning. For example, if one is building statistical models of patient outcome across a country, it might be natural to group together parameters of regions, cities, hospitals and individual doctors, corresponding to multiple levels of a hierarchy. Third, hierarchies provide good abstractions for interpretability of complex models. For example, rather than trying to understand what the hundreds of states in an HMM each do, it would be useful to have an HMM that partitions the states in a coarse to fine hierarchy so that one can start out by interpreting the coarse states and gradually move down to the fine states.

Note that while hierarchical clustering models are often described in terms of hierarchies over data points, in the above examples, we have seen that hierarchies can be useful more generally over internal components of models, such as hidden states of an HMM, or parameters of a model.

We have seen that the DPM model (and the associated CRP) can be used to define Bayesian non-parametric models for flat clustering. Are there equivalent Bayesian non-parametric models that result in hierarchical clusterings?

The answer is yes. Here, we very briefly touch upon two frameworks for generating hierarchies that can be used in non-parametric models. For the non-parametric setting, the key requirement is that the models define an infinitely exchangeable distribution over the data points. For this to hold, the models must be projective in the sense that marginalizing over the (*N*+1)th point should result in a coherent model over *N* points [57].

The first solution is given by *Kingman’s coalescent* [58]. This model has been widely used for modelling population genetics [59] and more recently in the machine learning community for hierarchical clustering [60]. The coalescent defines distributions over trees by starting with every data point in its own cluster and considering a process that merges clusters *backwards* in time until only one cluster remains. The key property of the coalescent is that for each pair of clusters the event that they merge is independent of the event that any other pair merges, and the time to this event is drawn from an exponential distribution with constant rate (which we can set to one without loss of generality). This process is well defined in the limit of 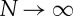 data points, and defines an infinitely exchangeable distribution over points.

A second solution is given by the Dirichlet diffusion tree (DDT; Neal [61]). Like Kingman’s coalescent, the DDT also defines a tree by considering a Markovian process evolving in time; however, the DDT starts at time *t*=0 and evolves *forward* for one unit of time.^11^ We denote the evolution of the *n*th point in time via *x*_*n*_(*t*). The first data point starts at a location *x*_1_(0)=0 and follows a Brownian motion process, ending up at some point drawn marginally from a unit variance Gaussian, *x*_1_(1)∼*N*(0,1). The second data point exactly follows the path of the first data point until a divergence event occurs, after which point its path is independent of the path of the first point. The time to divergence is parametrized through a *hazard function*, an object commonly used in survival analysis. Subsequent data points follow the paths of previous data points, diverging according to a scaled form of the hazard function, and when reaching a branch point choosing a branch with probability proportional to the number of points that chose that branch before. This process defines an exchangeable distribution over data points, parametrized by the unknown tree. Using the DDT prior, the problem of hierarchical clustering becomes one of inferring the unknown tree given some data.

Both Kingman’s coalescent and the DDT generate binary trees with probability one.^12^ A generalization of the DDT that allows arbitrary branching of the trees is given by the Pitman–Yor diffusion tree (PYDT; [62]). The process is generalized to allow, at each branch point, for the new data point either to follow the path of one of the previous points, or to create a new branch. Like the Pitman–Yor process [63], the PYDT has two parameters controlling its branching structure. Certain settings of these parameters result in the DDT, while other settings recover the distribution over trees induced by Kingman’s coalescent. The tree distribution induced by the PYDT is the multi-furcating Gibbs fragmentation tree [64], the most general Markovian exchangeable distribution over trees. General distributions over hierarchies and other clustering structures can also be derived through the elegant theory of fragmentation–coagulation processes [65].

## 9. Covariance matrices: generalized Wishart processes

Often we are interested in modelling the relationship between a number of variables. One can express relationships between variables in many different ways, but perhaps the simplest representation of dependence is the *covariance matrix*, *Σ*, a symmetric positive (semi)definite matrix representing second-order statistics of the variables. The covariance matrix plays a key role in parametrizing many distributions, most notably the multi-variate Gaussian, but also extensions such as the multi-variate t-distribution or more generally elliptical distributions [66].

In certain applications, we wish to model how such a covariance matrix might depend on some other variables. For example, in econometrics and finance, one is often interested in modelling a time-varying covariance matrix *Σ*(*t*)—this is the key object of interest in the field of multi-variate stochastic volatility [67]. More generally, we would like to place distributions on covariance matrices that can depend on arbitrary variables, *Σ*(*x*), not just scalar time. Viewed as a *function* of *x*, we want to be able to define distributions on matrix-valued functions restricted to the space of symmetric-positive-definite (s.p.d.) matrix values. Is there a convenient and simple way to define such a stochastic process?

Indeed there is, and the key insight comes from the observation that one can generate s.p.d. matrices by taking the outer products of random vectors. Consider the following construction, where we draw *D*-dimensional vectors independently from a multi-variate Gaussian distribution 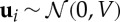 with covariance matrix *V* , and we define

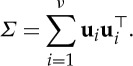

Such a matrix *Σ* is Wishart distributed with *ν* degrees of freedom and is s.p.d. with probability one as long as *ν*≥*D*. The mean of *Σ* is proportional to *V* .

We can generalize the Wishart distribution to a stochastic process indexed by *x* in any space 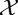 by replacing the elements of each ***u***_*i*_ with draws from a GP: ***u***_*i*_(*x*)=(*u*_*i*1_(*x*),*u*_*i*2_(*x*),…,*u*_*iD*_(*x*)), where

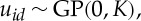

where *K* is the covariance function or kernel of the GP [21].

The desired stochastic process is obtained by the same construction

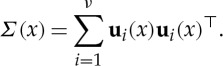

A special case of such a construction where the GPs are assumed to be Brownian has been studied in probability theory and is known as a *Wishart process* [68]; these processes have recently been applied to problems in econometrics [69,70]. The more general case for arbitrary GPs is developed in Wilson & Ghahramani [71] where applications to multi-variate stochastic volatility are also explored. In this general framework, the model parameters controlling *V* , *K* and *ν* can be learned from data.

## 10. Inference

So far we have focused on describing a general probabilistic approach to modelling and some non-parametric distributions that can be used in models of complex datasets. The three key ingredients in any approach to modelling are the data, the model and an *algorithm for doing probabilistic inference* in the model. The problem of probabilistic inference corresponds to computing the conditional probability of some variables of interest given some observed variables, while marginalizing out all other variables. Thus, both the computation of a model’s marginal likelihood (equation (2.1)) and prediction (equation (2.2)) are inference problems. Filling in missing data given observed data, computing the parameter posterior, or evaluating expectations of various quantities can all also be phrased as instances of the inference problem.

Generally, the inference problem boils down to a problem of numerical integration or summation over large state spaces. For most models of interest, especially non-parametric models, exact inference is computationally *intractable*, in the sense that all known algorithms for computing the exact probabilities of interest scale exponentially with some aspect of the problem, such as the number of data points or variables. In many problems, even approximating these exact probabilities to within some small error tolerance is intractable in the worst case.^13^

A wide variety of approximation methods have been developed to solve Bayesian inference problems. These can be roughly divided into stochastic approximations (which make extensive use of random numbers) and deterministic approximations. Some examples of widely used stochastic approximate inference methods include MCMC methods (for an excellent review, see Neal [72]), exact sampling methods [73] and particle filtering methods [74]. Some examples of deterministic algorithms include the Laplace approximation, variational methods [75] and expectation propagation [76]. Both deterministic and stochastic algorithms for inference can often exploit the conditional independence relationships that exist between the variables in a model to perform the relevant computations efficiently using local messages passed between nodes of a graphical model [77,78,79].

A complete review of approximate inference methods is beyond the scope of this paper, but a couple of points are worth making. All approximate inference methods can be characterized in terms of a speed–accuracy trade-off. Some methods are fast but often inaccurate, while other methods are slow or, such as MCMC, can be run for increasing amounts of time to give increasingly accurate results. There is no general rule of thumb for which approximate inference method is best—different models and problems tend to favour different methods. However, for a particular problem, the difference between a good choice of inference algorithm and a poor choice can be orders of magnitude of computation. Thus, it is well worth being familiar with a number of inference algorithms and being willing to try several methods on a particular problem.

The field of Bayesian statistics has thrived in recent years, both due to the availability of better inference algorithms and the dramatic growth in computing power. Ironically, the most widely used inference method in Bayesian statistics is MCMC, which itself is a thoroughly frequentist (non-Bayesian) method for approximating integrals. From a Bayesian perspective, numerical computation problems are also inference problems. In numerical integration, one is trying to infer the value of an integral by computing the integrand at a limited number of locations. The values of the integrand are the *data*, which, combined with a prior on the integrand, can result in a posterior on the value of the integral. The choice of where to evaluate the integrand can be made using Bayesian decision theory, clearly the random evaluations of MCMC are not an optimally efficient method for evaluating integrals. The Bayesian approach to numerical integration is known variously as Bayesian quadrature or Bayesian Monte Carlo, and although it is not as widely used as MCMC, it can be dramatically more efficient in situations where evaluating the integrand is computationally costly [80–83].

Deriving the approximate inference equations for each new model can be tedious and error prone, thereby inhibiting the researcher’s ability to explore many variations on any given model. The field of *probabilistic programming* offers an exciting alternative approach to building and evaluating models. Three notable examples of probabilistic programming frameworks are *BUGS*, *Church* and *Infer.NET* [84–86]. The basic idea is to write down a computer program defining the generative model in a programming language that is augmented to have random variables. This computer program that defines the generative model can then be *automatically* transformed or manipulated into a program that performs approximate posterior inference in the model. Thus, the process of deriving the approximate inference equations is automated, reducing the risk of human error. One potential disadvantage of this approach is that the automatically derived inference code may not be as efficient as an expertly designed inference method for a particular model. However, the rapid model development cycle that can result from the use of probabilistic programming languages offers tremendous advantages for the future of probabilistic modelling.

## 11. Conclusions

Modelling is fundamentally a process of quantifying uncertainty about possible predictions given available information. Probability theory provides an elegant framework for representing all sources of uncertainty in a model. Probability theory also provides the simple rules with which to manipulate models so as to obtain predictions, to compare models and to learn models from data. Bayesian statistics is simply the application of the rules of probability theory to modelling from data.

The probabilistic approach to modelling is most effective when the models are flexible enough to capture relevant aspects of the problem domain. Flexibility can be achieved by allowing models either to have many parameters, or in the limiting case, to have infinitely many parameters, in other words to be *non-parametric*. Learning models with infinitely many parameters may seem statistically and computationally daunting. However, statistically we have seen in §2 that the process of Bayesian averaging or marginalization avoids overfitting and therefore allows one to use models with infinitely many parameters (§3). Computationally, we have seen that there are a wide range of approximation methods that can be used to perform the relevant marginalizations required for inference (§10).

This article has attempted to give an overview of the basics of Bayesian modelling, the motivation for Bayesian non-parametrics and some of the more widely used non-parametric models. We have touched upon some computational issues, and briefly alluded to some successful applications of Bayesian non-parametrics. Of course, there is a great deal of current work in this rapidly advancing field that was not covered in this study.

There are four areas of future work worth highlighting: theory, new models, scalable algorithms and new applications. Regarding *theory*, developing frequentist consistency and convergence rate results for Bayesian non-parametric models is challenging but important. In particular, the effect of the prior on an infinite-dimensional space can result in inconsistent models in certain cases, so careful study of consistency is required.

While we have many building blocks for models, *new models* are an exciting area for work as they can lead to novel applications. General results on the construction of Bayesian non-parametric models, such as the recent work of Orbanz [57], are particularly useful for developing new models.

Many modern applications of statistical modelling and machine learning require the analysis of very large datasets (this is sometimes called ‘big data’). While a number of very good approximate inference methods have been developed in the last few decades, making these highly *scalable* is a challenge that requires extensive tools from computer science including efficient data structures, and parallel and distributed computing paradigms.

Finally, novel *applications* of Bayesian non-parametric modelling will advance the field in many ways. First, many new models are derived in response to the challenges arising from new applications. Second, applications can motivate general scalable inference solutions. Finally, successful applications will help in widening the adoption of a Bayesian non-parametric approach to modelling.

## Footnotes

1 With apologies to probabilists and statisticians, my presentation and notation will eschew formality in favour of didactic clarity.

2 Of course, our model may have a fixed value of *θ*, e.g. 7.213, which corresponds to a delta function prior.

3 Consider, for example, a model that defines the probability of the data (i.e. the likelihood) using a student *t*-distribution. For a single data point, this can be equivalently expressed as a model with a Gaussian likelihood and a Gamma prior on the precision of that Gaussian.

4 I do not like the term ‘random variable’ because it suggest that there is some true source of randomness driving some process. To give another example, people would naturally and understandably flinch at the idea of calling something like the millionth digit of *π* or the number of people alive today a random variable. The notion that Bayesians consider true parameters to be ‘random variables’ makes the framework less intuitive than it actually is. It is much more natural to use the term *uncertain*. Clearly I can be uncertain about the millionth digit of *π*, even though its value is not random at all. My uncertainty about this digit will change after I look it up on the web, but presumably will not go to zero as there is always the chance of someone having made a mistake somewhere.

5 To the best of my knowledge, this information channel view of non-parametric modelling is not explicitly articulated in the literature, although to be sure, there are a number of strong links between Bayesian inference and information theory [8,18]. These ideas could certainly be further formalized by explicitly attempting to measure or estimate the channel capacity of different models. This would help provide a further definition of the *complexity* of a model, measured in bits per data point, which in many ways is more satisfactory than definitions that rely on counting parameters.

6 There are generalizations of exchangeability to handle time series and arrays.

7 The word ‘process’ usually evokes the idea of something evolving temporally, but it is important to note that stochastic processes can be indexed by time, space or any other index space. Many of the uses of stochastic processes in Bayesian non-parametrics do not correspond to indexing by time.

8 There is a subtle but important distinction between assuming an infinite model, and assuming a finite but potentially unbounded model. The difference comes to light when we consider what we expect to happen in the limit of infinitely large datasets. In the former case, the posterior will have visited infinitely many distinct states, while in the latter case, the posterior should converge on some finite number of states.

9 The beam sampler is available in the software package http://mloss.org/software/view/205/.

10 It is not hard to reintroduce the second parameter to get the two-parameter IBP.

11 Note that both in the coalescent and in the DDT, ‘time’ is an indexing variable used to generate a tree structure, and need not correspond to a real notion of time.

12 In the case of the DDT, binary trees result in all but pathological cases where the hazard function diverges before *t*=1.

13 Most theoretical results on intractability focus on the worst case of a problem instance. The real-world inference problem may in fact be much easier to approximate.
